# Seed rain and soil seed bank in Chinese fir plantations and an adjacent natural forest in southern China: Implications for the regeneration of native species

**DOI:** 10.1002/ece3.8539

**Published:** 2022-01-26

**Authors:** Bo Liu, Qingqing Liu, Chenxi Zhu, Zhigang Liu, Zhijun Huang, Mulualem Tigabu, Zongming He, Yuhui Liu, Zhengning Wang

**Affiliations:** ^1^ College of Life Sciences Qufu Normal University Qufu China; ^2^ 12449 College of Forestry Fujian Agriculture and Forestry University Fuzhou China; ^3^ Forestry Bureau of Xinxiang Xinxiang China; ^4^ 8095 Southern Swedish Forest Research Center Faculty of Forest Science Swedish University of Agricultural Sciences Alnarp Sweden; ^5^ 12449 Xinkou Teaching Forest Farm Fujian Agriculture and Forestry University Sanming China

**Keywords:** afforestation, conifers, ecological restoration, mixed plantation, Sørensen similarity index, subtropical forests

## Abstract

The natural regeneration of native broadleaved species underneath forest monoculture plantations is important to recover ecosystem functions and to mitigate adverse environmental effects. To understand how seed rain and soil seed bank facilitate natural regeneration, we surveyed their density and composition in a monoculture Chinese fir plantation, a mixed Chinese fir–broadleaf plantation, and an adjacent natural broadleaved forest for two years in southern China. Twenty‐eight species (16 families) were in seed rain, and 45 species (27 families) were in soil seed bank. Seed rain density did not differ significantly across stands; however, the number of taxa in seed rain was highest in the mixed plantation and lowest in the natural forest. Seed bank density was significantly higher in the mixed plantation than in the other stands (*p* < .05). The Sørensen similarity index of species composition between seed sources and aboveground vegetation were relatively low (<.50). The seeds of various native tree species were common in the seed bank of the plantations, indicating that seed rain and seed bank played an important role in native forest regeneration. We recommend that managers interested in sustainable forestry should take into consideration the presence of existing soil seed bank when developing their management strategies. In addition, with regard to forest regeneration process, we also recommend supplementation of the species composition by direct seeding or planting of desired species.

## Graphical abstract

Our findings demonstrated that seeds from native tree species in the natural forest were present in the plantation seed rain and seed banks. Furthermore, species composition differed across seed rain, seed banks, and aboveground vegetation. The two seed sources are valuable for restoring species diversity in plantations and achieving sustainable forest management.

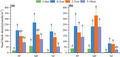

## 1 INTRODUCTION

Plantations are replacing natural forests worldwide because of economic growth and the resultant high demand for timber (Payn et al., 2015). In 2015, the total plantation area reached 278 million ha, which was 6.95% of global forests (Keenan et al., 2015; Payn et al., 2015). With its rapidly developing economy, China has not been exempt from the past five decades of change (CSFB, 2014), and the nation currently has the largest plantation area in the world, with over 69 million ha, which is 25% of total plantations (CSFB, 2014; Payn et al., 2015). Natural forests are complex adaptive systems dominated by native tree species that regenerate themselves naturally, with uneven age and longevity (Yang et al., 2018). In contrast to natural forests, pure plantations generally comprise one artificially established tree species, whose individuals are even aged and regularly spaced (Payn et al., 2015). Many pure plantations also comprise genetically improved native tree species or fast‐growing exotic species. At the same time, pure plantations have high stem density, high intensity of human interventions, and continuous planting. So, the pure plantations have experienced several serious problems compared to natural forests, such as degrading biodiversity, soil fertility, ecosystem functioning and services, as well as limiting natural regeneration (Chen et al., 2017; Gardiner et al., 2009; Richards et al., 2010). This problem is of particular concern when plantations are monocultures of fast‐growing pioneer species, as is the case in southern China (CSFB, 2014; Yang et al., 2018). To improve the sustainability of these forest monocultures, silvicultural practices based on optimal forest management strategies are urgently needed. Forest sustainability is based on adequate forest management strategies. The implementation of silvicultural practices in forest management has to guarantee forest sustainability, which is supported by adequate tree regeneration. Also, natural regeneration has been viewed as an effective forest management strategy with a low‐cost, effective, and sustainable strategy that is consistent with the modern emphasis on protecting biodiversity (Bertacchi et al., 2016; Chazdon, 2008). Thus, a major objective in forest management research is to identify approaches that can accelerate plantation succession to natural forest and promote establishment of native species (Onaindia et al., 2013; Yan, Zhu, & Gang, 2013, Yan, Zhu, Gang, Huang, et al., 2013).

Chinese fir [*Cunninghamia lanceolata* (Lamb.) Hook; Taxodiaceae] is a major afforestation species in southern China, accounting for 24% (take up 17 million ha) of the nation’s forest plantations and 6.1% of those worldwide (Liu et al., 2018; Payn et al., 2015). This species is fast‐growing, high‐yielding, has excellent wood quality (Duan et al., 2016; Li, 1992), and plays an important role in environmental protection (Ma et al., 2007; Yang et al., 2018). Most Chinese fir plantations occur on sites formerly dominated by natural broadleaved forests containing species such as *Castanopsis kawakamii* Hayata., *Castanopsis carlesii* (Hemsl.) Hayata., and *Schima superba* Gardn. et Champ (He et al., 2012). Unfortunately, long‐standing unsustainable management and overexploitation have caused regeneration problems for native broadleaved species in Chinese fir plantations, endangering long‐term forest productivity (Chen et al., 2014; Luo et al., 2014; Ma et al., 2007; Yang et al., 2004; Zhu et al., 2010 **)**. The interest in addressing this problem has increased as the focus of forest management shifted from timber production to sustainable forest management around the end of the last century (Yan, Zhu, & Gang, 2013, Yan, Zhu, Gang, Huang, et al., 2013; Yang et al., 2018).

In many ecosystems, natural regeneration relies on seed rain and soil seed bank (Augusto et al., 2001; Boudell & Stromberg, 2008; Douh et al., 2018; Moles & Drake, 1999; Ssali et al., 2018). Seed rain results from seed production within a plant community and seed input from adjacent communities (Douh et al., 2018). Seed rain plays a key role in the subsequent recruitment of new plants and, thus,determines forest community structure, dynamics, and regeneration (Fuller & Moral, 2003; Pakeman & Small, 2010; Tackenberg & Stocklin, 2008). Seed bank reflects the composition of current and past plant communities (Bossuyt et al., 2002). Both seed rain and soil seed bank buffer against environmental instability by providing new seedlings to re‐establish the native community after disturbances, with soil seed bank being particularly valuable for long‐lived species (Augusto et al., 2001). Furthermore, seed rain and seed bank are sources of colonizing species that can restore degraded ecosystems and accelerate forest succession (Douh et al., 2018; Hubbard & McPherson, 1999; O’Donnell et al., 2016). Consequently, information on composition and size of seed rain and seed bank has practical importance in forestry because it is critical to understand the natural regeneration mechanisms in both native forests and plantations. The lack of appropriate seed sources is the primary bottleneck limiting native species colonization of plantations (Barbosa & Pizo, 2006; Denslow et al., 2006; Wang, Zou, et al., 2009). However, some types of plantations inherently increase the regeneration of native understory species and contribute to biodiversity conservation (Augusto et al., 2001; Lemenih & Teketay, 2005; Onaindia et al., 2013; Yan, Zhu, & Gang, 2013, Yan, Zhu, Gang, Huang, et al., 2013), which is likely due to adequate seed rain or seed bank containing succession‐accelerating species (Augusto et al., 2001; Hubbard & McPherson, 1999).

In this study, we evaluated the contributions of seed rain and soil seed bank to the natural regeneration of native species in cultivated Chinese fir stands. We compared the composition and size of seed rain and soil seed bank in a monoculture Chinese fir plantation, a mixed Chinese fir–broadleaf plantation, and an adjacent natural forest. Specifically, we addressed the following questions: (1) How does the species composition of seed rain and soil seed bank differ across forest stands? (2) Does the species composition of seed rain and seed bank reflect aboveground vegetation? Our findings will help determine whether unaided natural regeneration is sufficient to restore primary native forests or intervention is needed. Thus, our results should be useful in planning appropriate management strategies for succession of mixed‐forest within Chinese fir plantations.

## 2 MATERIALS AND METHODS

### 2.1 Study site

The study site was in the Xinkou Experimental Forestry Centre of Fujian Agricultural and Forestry University (26°19′N, 117°36′E), Sanming City, Fujian Province, China. The regional climate is typical maritime subtropical monsoon with mean annual rainfall of 1670 mm (approximately 80% occurring between March and August) and mean annual temperature of 20.1°C (data from 1959 to 2006) (Yang et al., 2018). The soil is lateritic. Before 1957, natural forests covered the entire study area. Subsequently, large portions were clear cut and replaced with monoculture and mixed plantations, resulting in three types of stand communities for conservation. Field observations were conducted in the following: (1) a monoculture Chinese fir plantation (PP), (2) a mixed Chinese fir–*S. superba* plantation (i.e., mixed conifer–broadleaf plantation, MP), and (3) a natural forest (NF) adjacent to the plantations. Typical of the subtropics, *C. kawakamii* (recalcitrant seeds), *C. carlesii* (recalcitrant seeds), and *S. superba* dominated the evergreen broadleaf NF (He et al., 2012). All selected stands were from 205 to 215 m.a.s.l. The topographic and climatic microhabitat characteristics of the different stand types were assumed to be similar because they were adjacent to one another.

### 2.2 Seed rain and seed bank sampling

Seed rain was measured using seed traps. Within each stand, five parallel transects (at least 10 m apart) were established, running from the lower to the upper slope. Nine seed traps were set up at 10‐m intervals along each transect. Therefore, there were 45 seed traps (nine seed traps per transect × 5 transects) in each stand. Seed traps were made of cubical PVC tube frames and vinylon cloth (mesh size: 1 mm), supported by four PVC tubes. Each seed trap was 15 cm deep, and its base was positioned 20 cm above the ground, and the area of each seed trap was 1.0 m × 1.0 m. The seed traps remained open throughout the study period, with seeds, fruits, and seed‐bearing fruits collected monthly from February 2016 to February 2017 (first year) and from March 2017 to February 2018 (second year). Collected materials were transported to the laboratory, sorted by species, and counted. The seed extraction method (Douh et al., 2018; Yan et al., 2016) was used to determine the amount and composition of seed rain. Identification was based primarily on comparison with seeds obtained from plants growing in the surrounding area. Species were classified into three growth forms (trees, shrubs and vines, and herbs) and two functional groups (pioneer species and shade‐tolerant species). Because the seed traps were set 20 cm aboveground, they largely collected post‐dispersal seeds and fruits. Although this method may underestimate seed rain density, it can precisely represent the post‐dispersal state of seeds and has been widely applied in studies of forest ecosystems (Douh et al., 2018; Pakeman & Small, 2009; Wang, Ren, et al., 2009).

Soil seed bank samples (45 per stand) were collected in March 2017 and March 2018 from the same positions as the seed rain traps. Forest litter and three soil depths (0–2, 2–5, and 5–10 cm) were sampled at each sampling point. The surface area of each sample was 400 cm^2^ (20 cm × 20 cm). Seed viability in the soil seed bank was estimated using the seedling emergence method because it can determine the viable fraction of readily germinable seeds (Douh et al., 2018; Sousa et al., 2017; Wang et al., 2013). Despite the potential to underestimate seed bank density, the seedling emergence method remains the most common and feasible technique in soil seed bank studies (Douh et al., 2018; Sousa et al., 2017). And, this method allowed us to provide a good perspective for managers in using in situ seed bank as opposed to sowing seed as source material for vegetation restoration efforts. Each soil sample was distributed on a 5‐cm deep perlite layer in a seed germination tray, following the method of Wang et al. (2013), with soil depth maintained at approximately 1.0 cm. In order to keep soil depth maintained at approximately 1.0 cm, the size of seed germination traps was 20 cm × 40 cm × 8 cm, 30 cm × 40 cm × 8 cm, and 50 cm × 40 cm × 8 cm for 0–2 cm, 2–5 cm, and 5–10 cm soil layer sampling, respectively. The seed germination trays were placed in an experimental greenhouse where temperature varied from 15°C to 35°C, closely resembling the study area’s seasonal temperatures. Seed germination trays were watered daily to maintain soil moisture. Every 5 days, newly germinated seedlings were identified to species, counted, and then removed from trays. Unknown seedlings were transplanted into larger pots for further growth until the species could be identified. Germination was monitored for 6 months until no further seedlings emerged for three weeks, consistent with previous studies adopting a 4–6‐month germination time frame (Douh et al., 2018; Savadogo et al., 2017). During the six months, soil samples were turned over five times to unearth seeds that had not germinated, thereby potentially increasing seed exposure to light. In our study, the total number of emerged seedlings was used as a measure of viable seeds in seed bank.

### 2.3 Aboveground vegetation survey

Plant species composition and density were assessed per stand in order to compare the species in soil seed bank or seed rain with those in the aboveground vegetation. Along the seed rain/seed bank transects, trees were sampled in six 10 m × 10 m quadrats. Within each quadrat, two 5 m × 5 m shrub quadrats and four 1 m × 1 m herbaceous plant quadrats were also sampled. The species, cover, and height were recorded in each quadrat. Vegetation surveys were conducted in July 2017.

### 2.4 Data analyses

Seed rain density (seeds m^−2^) was calculated from the number of seeds captured in each seed trap. Soil seed bank density (seedlings m^−2^) was calculated from the number of emerged seedlings. The Sørensen similarity index (SI) was used to compare aboveground and seed rain or seed bank species composition in each stand (Sokal & Sneath, 1963):

$$\text{SI}=\frac{2c}{\left(a+b\right)}$$

where c is the number of common species between the seed rain/seed bank and the aboveground community, a is the total number of species in the seed rain/seed bank, and b is the total number of aboveground species. All data were tested for homogeneity of variance and subjected to one‐way ANOVA to determine significant between‐stand differences in mean densities of seed rain and seed bank. Within each stand, Student’s *t*‐test was used to distinguish between the two years of data. Statistical significance was set at *p* < .05. Analyses were performed in SPSS 21 for Windows.

## 3 RESULTS

### 3.1 Seed rain

**FIGURE 1:**
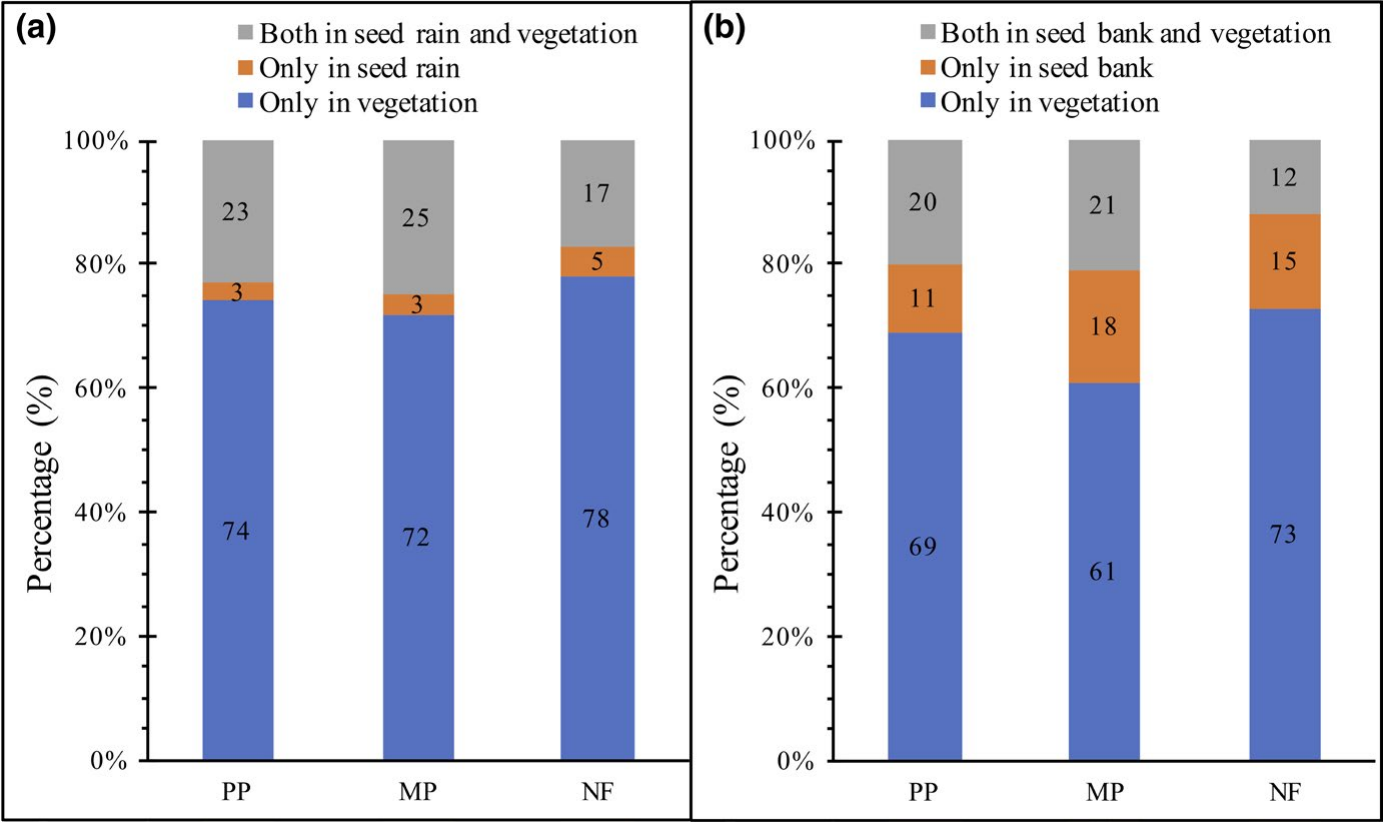
Percentages of species found only in seed rain, only in soil seed bank, only in aboveground vegetation, and in both seed rain/soil seed banks and aboveground vegetation in three subtropical forest stands in southern China. Monoculture Chinese fir plantation (PP), mixed Chinese fir–broadleaf plantation (MP), and natural broadleaf forest (NF)

**FIGURE 2:**
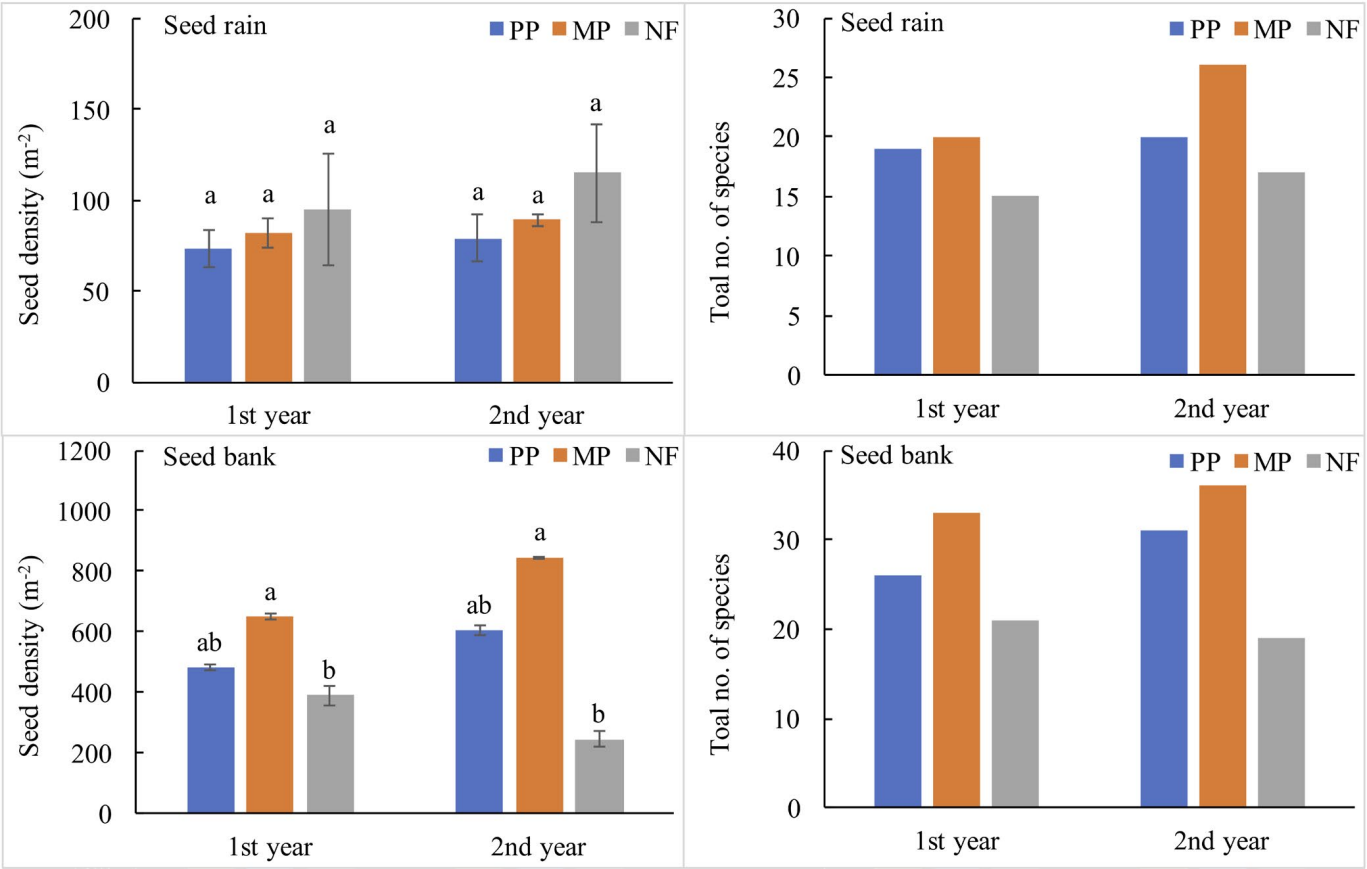
Seed density (seeds m^−2^) and total number of species in seed rain and in soil seed banks in a monoculture Chinese fir plantation (PP), a mixed Chinese fir–broadleaf plantation (MP), and a natural broadleaf forest (NF) over two years in southern China. Different lowercase letters indicate significant differences (*p* < .05) between stands. No significant differences were detected between years

We collected 24,823 seeds (28 species and 16 families) in seed rain from the three stands (Table 1). Approximately 17% to 25% of the species were common to seed rain and aboveground vegetation, whereas 3% to 5% were found only in seed rain, and 72% to 78% were found only in the aboveground vegetation of the three stands (Figure 1). The species common in seed rain across the three stands were *C. lanceolata*, *S. superba*, *Pinus massoniana* Lamb., *Daphniphyllum oldhami* (Hemsl.) K. Rosenthal, *Mallotus lianus* Croiz., *C. carlesii*, *C. kawakamii*, *Neolitsea aurata* var. *chekiangensis*, and *Machilus pauhoi* Kanehir*a* (Table 1). The dominant species in seed rain of pure plantation (PP) and mixed plantation (MP) was *C. lanceolata*, representing 50% (first year) and 57% (second year) of total seeds in the PP and 28% of total seeds in both years in the MP. *S. superba* was dominant in seed rain of natural forest (NF), accounting for 75% (first year) and 84% (second year) of total seeds. Tree species dominated in seed rain, representing 87% to 99% of all seeds. The most common seed‐dispersal modes were zoochory, representing 47% to 65% of the species in the seed rain over the two years, and anemochory, representing 12% to 20% of the species. Across all stands, seed rain density did not exhibit temporal variation, ranging from 73 to 95 seeds m^−2^ y^−1^ in the first year and from 79 to 115 seeds m^−2^ y^−1^ in the second year (Figure 2). The number of seed taxa was highest in the MP and lowest in the NF (*p* < .05) (Figure 2).

**TABLE 1:** Species composition, functional group (P, pioneer; ST, shade‐tolerant), dispersal mode, and number of seeds in the seed rain of a monoculture Chinese fir plantation (PP), a mixed Chinese fir–broadleaf plantation (MP), and a natural broadleaf forest (NF) over two years in southern China

Species	Family	Functional group	Dispersal mode	PP		MP		NF	
				1^st^ year	2^nd^ year	1^st^ year	2^nd^ year	1^st^ year	2^nd^ year
Trees									
*Cunninghamia lanceolata*	Taxodiaceae	P	Anemochory	933	1014	2108	2039	32	20
*Schima superba*	Theaceae	ST	Anemochory	565	2111	573	1374	3429	4706
*Pinus massoniana*	Pinaceae	P	Anemochory	13	8	73	24	280	25
*Symplocos sumuntia*	Symplocaceae	ST	Zoochory	14	7	0	2	0	5
*Diospyros morrisiana*	Ebenaceae	ST	Zoochory	6	0	40	1	0	0
*Elaeocarpus sylvestris*	Elaeocarpaceae	ST	Zoochory	0	1	0	1	0	1
*Daphniphyllum oldhami*	Daphniphyllaceae	ST	Zoochory	13	1	28	5	12	4
*Toxicodendron succedaneum*	Anacardiaceae	ST	Zoochory	0	4	2	12	17	24
*Choerospondias axillaris*	Anacardiaceae	P	Gravity	0	0	0	38	0	0
*Vernicia montana*	Euphorbiaceae	P	Gravity	0	0	0	16	0	0
*Mallotus lianus*	Euphorbiaceae	ST	Gravity	9	51	33	73	11	63
*Castanopsis carlesii*	Fagaceae	ST	Gravity	611	87	222	100	316	446
*Castanopsis kawakamii*	Fagaceae	ST	Gravity	66	0	30	5	108	247
*Lithocarpus glaber*	Fagaceae	P	Gravity	2	12	2	0	0	0
*Litsea subcoriacea*	Lauraceae	ST	Zoochory	2	3	14	1	0	0
*Neolitsea aurata* var. *chekiangensis*	Lauraceae	ST	Zoochory	397	159	134	129	15	1
*Neolitsea cambodiana* var. *glabra*	Lauraceae	ST	Zoochory	33	0	3	28	19	0
*Machilus pauhoi*	Lauraceae	ST	Zoochory	196	49	148	20	210	2
*Phoebe zhennan*	Lauraceae	ST	Zoochory	2	1	11	2	8	2
*Machilus thunbergii*	Lauraceae	ST	Zoochory	0	4	0	6	0	0
Shrubs and vines									
*Ardisia punctate*	Myrsinaceae		Zoochory	0	0	0	77	0	0
*Embelia longifolia*	Myrsinaceae		Zoochory	7	2	104	67	2	0
*Camellia cuspidate*	Theaceae		Gravity	0	0	0	12	0	0
*Litsea cubeba*	Lauraceae		Zoochory	406	15	161	24	7	5
*Millettia reticulate*	Fabaceae		Ballistic	8	1	5	2	90	4
*Diploclisia affinis*	Menispermaceae		Zoochory	0	6	0	17	0	3
*Kadsura longipedunculata*	Magnoliaceae		Zoochory	0	0	1	0	0	0
Herbs									
*Alpinia chinensis*	Zingiberaceae		Zoochory	3	31	12	31	0	46
Total				3286	3567	3704	4106	4556	5604

### 3.2 Soil seed bank

**FIGURE 3:**
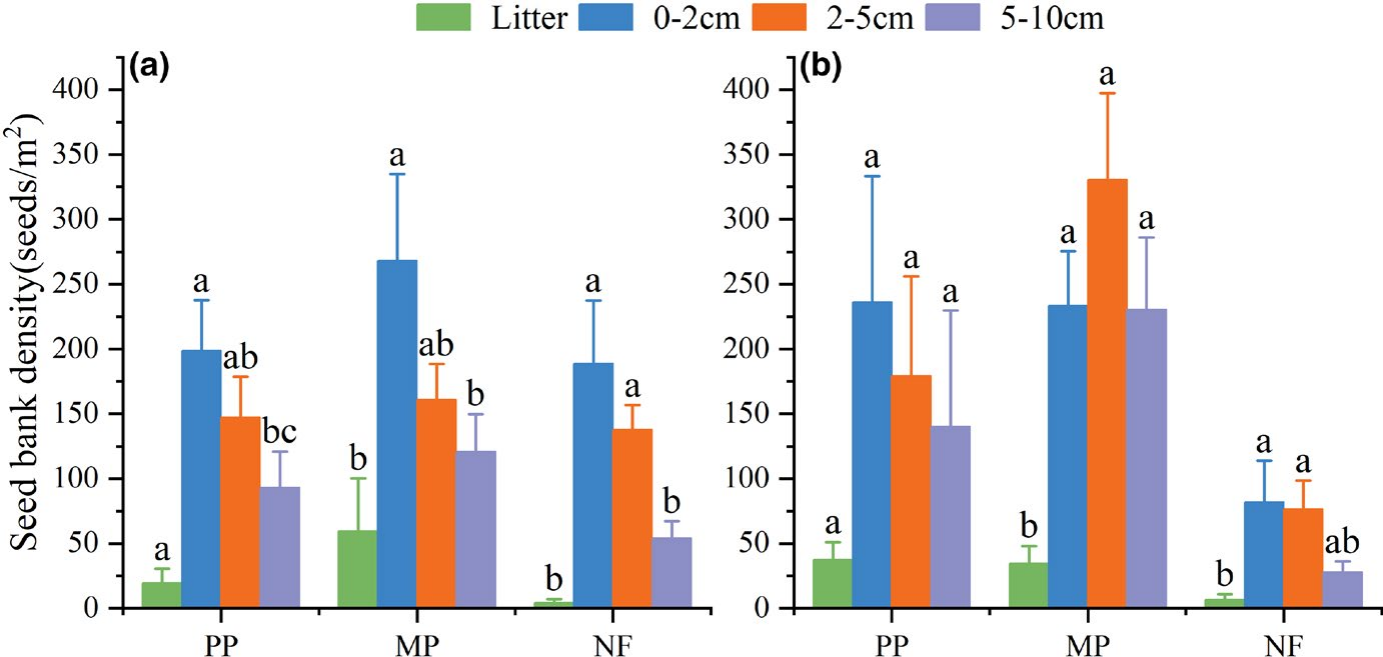
Vertical distribution of seed density (seeds m^−e^) in soil seed banks of a monoculture Chinese fir plantation (PP), a mixed Chinese fir–broadleaf plantation (MP), and a natural broadleaf forest (NF) in two consecutive years in southern China. (a) year one (2016–2017), (b) year two (2017–2018). Different letters indicate significant differences between soil layers in the same stand (*p* < .05)

In total, 57,655 seedlings (45 species and 27 families) were recorded from all soil seed bank samples (Table 2). Across all stands, 12% to 21% of the species were common to seed banks and aboveground vegetation, whereas 11% to 18% were unique to seed banks, and 61% to 73% were unique to aboveground vegetation (Figure 1). Soil seed density was significantly different across stands (Figure 2) and was highest in the MP (first year: 650 seeds m^−2^; second year: 847 seeds m^−2^), followed by the PP (first year: 482 seeds m^−2^; second year: 606 seeds m^−2^), and then the NF (first year: 390 seeds m^−2^; second year: 246 seeds m^−2^). However, seed density gradually decreased with increasing soil depth, excluding the litter layer (Figure 3). Specifically, in all stands across both years, the viable seed count was significantly higher in the 0–2‐cm layer than in the 5–10‐cm layer (*p* < .05), except for the lack of any difference in the MP during the second year (Figure 3).

**TABLE 2:** Species composition and seedling count in soil seed banks of a monoculture Chinese fir plantation (PP), a mixed Chinese fir–broadleaf plantation (MP), and a natural broadleaf forest (NF) over two years in southern China (1^st^ year, February 2016 to February 2017; 2^nd^ year, March 2017 to February 2018)

Species	Family	PP		MP		NF	
		1 year	2 year	1 year	2 year	1 year	2 year
Trees							
*Cunninghamia lanceolata*	Taxodiaceae	350	125	975	450	25	20
*Schima superba*	Theaceae	250	350	125	225	225	400
*Diospyros morrisiana*	Ebenaceae	20	0	50	25	0	0
*Elaeocarpus sylvestris*	Elaeocarpaceae	0	50	0	75	0	0
*Daphniphyllum oldhami*	Daphniphyllaceae	25	20	20	25	25	20
*Sapium discolor*	Euphorbiaceae	125	100	50	50	50	0
*Alangium kurzii*	Alangiaceae	125	25	75	50	0	0
*Mallotus lianus*	Euphorbiaceae	0	0	0	0	25	25
*Helicia cochinchinensis*	Proteaceae	650	675	150	75	0	0
*Trema tomentosa*	Ulmaceae	200	150	1175	875	750	150
*Machilus pauhoi*	Lauraceae	325	2150	825	1575	625	450
*Litsea greenmaniana*	Lauraceae	75	125	50	75	0	0
*Castanopsis carlesii*	Fagaceae	50	150	125	75	225	200
*Lithocarpus glaber*	Fagaceae	10	25	20	0	0	0
*Toxicodendron succedaneum*	Anacardiaceae	50	0	125	0	25	0
*Choerospondias axillaris*	Anacardiaceae	0	0	0	75	0	0
Shrubs and vines							
*Melastoma dodecandrum*	Melastomataceae	50	350	25	475	0	75
*Litsea cubeba*	Lauraceae	0	0	50	50	0	25
*Mallotus apelta*	Euphorbiaceae	200	75	125	75	175	25
*Rhododendron henryi*	Ericaceae	2025	125	1675	275	1100	0
*Callicarpa kochiana*	Verbenaceae	0	350	0	1225	0	50
*Clerodendrum canescens*	Verbenaceae	0	0	0	50	0	0
*Rubus reflexus*	Rosaceae	0	100	25	200	0	900
*Rubus corchorifolius*	Rosaceae	0	100	0	0	0	0
*Maesa japonica*	Myrsinaceae	0	650	0	450	0	25
*Embelia longifolia*	Myrsinaceae	0	0	0	75	0	0
*Diploclisia affinis*	Menispermaceae	0	0	25	0	0	0
*Aristolochia obliqua*	Aristolochiaceae	175	150	200	175	25	50
*Ampelopsis grossedentata*	Vitaceae	0	0	25	75	50	0
Herbs							
*Alpinia chinensis*	Zingiberaceae	175	125	125	0	125	25
*Lygodium japonicum*	Lygodiaceae	25	2275	1125	2000	75	0
*Dennstaedtia wilfordii*	Dennstaedtiaceae	0	350	0	75	0	0
*Stenoloma chusanum*	Lindsaeaceae	700	250	475	1300	0	975
*Geranium wilfordii*	Geraniaceae	575	100	775	25	375	375
*Blechnum orientale*	Blechnaceae	0	700	0	1425	25	0
*Oplismenus undulatifolius*	Poaceae	0	0	275	150	0	0
*Echinochloa hispidula*	Poaceae	0	0	75	0	0	0
*Digitaria fibrosa*	Poaceae	300	350	25	1200	0	0
*Lophatherum gracile*	Poaceae	0	350	0	900	0	0
*Ageratum conyzoides*	Asteraceae	500	0	800	0	1750	0
*Conyza japonica*	Compositae	250	0	825	25	0	0
*Gahnia tristis*	Cyperaceae	325	325	300	1175	450	175
*Cyperus rotundus*	Cyperaceae	1125	100	550	325	1200	50
*Adiantum capillus‐veneris*	Adiantaceae	0	0	75	0	25	0
*Adiantum flabellulatum*	Adiantaceae	0	75	0	50	0	0
Total		8680	10845	11340	15425	7350	4015

Soil seed bank contained between 19 and 36 species, with the lowest numbers of species in the NF (Figure 2). Tree species accounted for 24% to 36% of the total number of seeds in seed bank, whereas herbaceous species accounted for 41% to 56% (Table 1). Six tree species were common to soil seed bank in all stands. The seeds of many tree species, such as *S. superba* and *C. lanceolata*, were found only in the litter layer. The seeds of herbaceous species were more abundant in the lower than in the upper soil layer.

### 3.3 Comparisons of species composition in aboveground vegetation, seed rain, and soil seed bank

**FIGURE 4:**
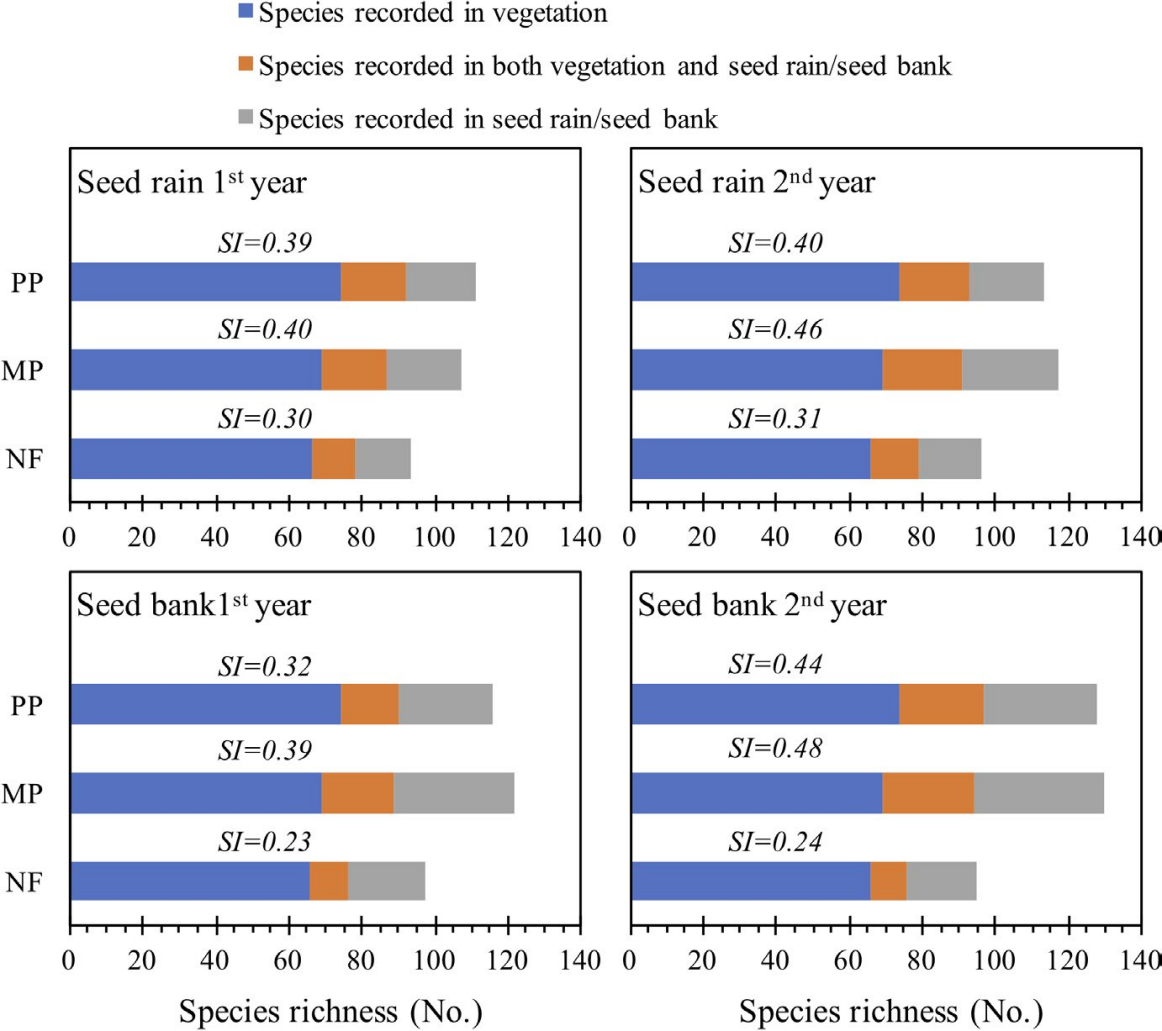
Species richness and similarity (SI, Sørensen similarity index) between species in aboveground vegetation and seed rain or seed bank in three forest stands in southern China. Monoculture Chinese fir plantation (PP), mixed Chinese fir–broadleaf plantation (MP), and natural broadleaf forest (NF)

The total number of species in aboveground vegetation ranged from 66 to 74 (Figure 4). The aboveground vegetation contained 76% to 95% of the species in seed rain and 50% to 73% of the species in seed bank (Tables 1 and 2, respectively). The Sørensen index (SI) between seed rain and aboveground vegetation ranged from 0.30 to 0.46, with the lowest and highest values in the NF and MP, respectively (Figure 4). Additionally, the SI between soil seed bank and aboveground vegetation ranged from 0.23 to 0.49, with the lowest values in the NF and the highest value in the MP (Figure 4).

## 4 DISCUSSION

Soil seed density in this study was similar to previously reported values for established conifer plantations in China: 220 seeds m^−2^ y^−1^ in a pure conifer plantation (Zheng et al., 2004), 967 seeds m^−2^ y^−1^ in a mixed conifer–broadleaf plantation (Wang, Ren, et al., 2009), 839 seeds m^−2^ y^−1^ in a *Robinia pseudoacacia* plantation (Wang & Ren, 2004), and 649 seeds m^−2^ y^−1^ in a *Larix principis rupprechtii* plantation. Compared with the natural forest in the same region, the monoculture Chinese fir and mixed conifer–broadleaf plantations had higher seed density and species richness. This result is consistent with that of previous studies demonstrating that managed forests have greater numbers of seeds and higher species richness than unmanaged forests (Godefroid et al., 2006; Wang, Ren, et al., 2009; Zobel et al., 2007).

Similar to previous findings (Daïnou et al., 2011; Douh et al., 2018; Grombone‐Guaratini & Rodrigues, 2002; Wang, Ren, et al., 2009), herbaceous species dominated soil seed bank in all three forest stands, varying between 43% and 56% of the total species. Moreover, the 5–10‐cm soil layer contained more herbaceous seeds than the 0–2‐cm and 2–5‐cm soil layers, and no herbaceous seeds were found in the litter layer. There are several possible explanations for the high densities of herbaceous seeds in soil seed bank. First, herbaceous species tend to produce many small seeds that are more likely to escape predation than larger seeds (Bekker et al., 2010; Moles & Westoby, 2010). Second, herbaceous seeds may persist longer than other seed types in seed bank, given the negative correlation between seed size and longevity (Dalling & Brown, 2009; Thompson et al., 1993). Third, the shaded understory conditions in mature plantations can inhibit germination of light‐requiring herbs (Daïnou et al., 2011).

Several other studies also showed that soil seed bank composition changes with soil depth (Bekker et al., 2010; Douh et al., 2018; Moles & Westoby, 2010). In this study, species richness and seed density were higher in the upper soil layer than in the lower soil layers. This pattern is most likely attributable to seed size (Luzuriaga et al., 2005) and vertical seed movement (Decocq et al., 2004). Our findings support the hypothesis that the large seeds of tree species are found mainly in the upper soil layer, whereas smaller herbaceous seeds are predominant in the lower soil layers (Bekker et al., 2010).

Despite differences in seed abundance, many species were common to soil seed bank in the three forests. Notable examples included seeds of native tree species (e.g., *S. superba*, *C. carlesii*, *D. oldhami*, *Sapium discolor*, *Trema tomentosa*, *M. pauhoi*, and *Litsea greenmaniana*). Thus, the adjacent natural forest appeared to function as a seed source for the plantations, providing native tree propagules for colonization. As colonizing native tree species develop, they gradually ameliorate unfavorable ecological conditions in plantations and facilitate succession to native mixed forest. Our work corroborates the idea that native forest fragments near plantations are necessary to maintain adequate seed sources for restoration (Moles & Drake, 1999). Thus, by leaving fragments of native forest as seed sources, plantation managers can promote natural succession to meet restoration objectives.

Our study indicated that the species composition of seed rain largely reflected the local plant community because a large percentage of the species identified in seed rain corresponded with those in aboveground vegetation (76% to 95%). However, many biotic and abiotic factors contribute to seed mortality, including seed predation, competition, and soil moisture deficiency (Au et al., 2006; Booth & Larson, 1998; Lenz & Facelli, 2005). Such factors can vary considerably over time, causing major shifts in the species composition of seed rain. Temporal fluctuations also occur in seed production and dispersal (Tackenberg & Stocklin, 2008), thereby altering the migration rate of a given species (Fuller & Moral, 2003). Therefore, our two years of data may not be fully representative of the typical seed rain in the three stands. Nevertheless, our study indicates that seed rain is most likely an important contributor to native broadleaf colonization of pure Chinese fir plantations.

Although the seed bank sampling area (0.04 m^2^) was only 4% of the seed rain sampling area (1 m^2^), mean seed density was higher in the seed bank than in seed rain, consistent with patterns observed in previous studies (Dupuy & Chazdon, 2008; Igarashi & Kiyono, 2008; Pakeman & Small, 2010). The difference is most likely a reflection of the difference between a single year of data for seed rain and data accumulated from multiple previous seed rains for soil seed bank. Furthermore, soil seed bank contains seeds of some species that persist in soil (Bekker et al., 2010), whereas the fresh seeds in seed rain are subjected to seed predation (Hubbard & McPherson, 1999). Seed rain and soil seed bank also show considerable annual fluctuation (Wang et al., 2010). In addition, the aerial traps likely missed some herbaceous seeds, thereby underestimating seed density. Species richness was also considerably higher in soil seed bank than in seed rain (Tables 1 and 2, and Figure 2). This result was most likely due to the greater number of herbaceous and shrub species in the seed bank, whereas the seeds of tree species were primarily found in seed rain. Furthermore, the species composition of recent seed rain did not adequately reflect soil seed bank composition at a given site.

In all three stands, the aboveground vegetation generally comprised species distinct from those in seed rain or soil seed bank. Approximately, 77%, 74%, and 86% of the aboveground taxa found in the vegetation did not occur in soil seed bank of the PP, MP, and NF stands, respectively (Appendix S1–3); on the other hand, 35%, 45%, and 57% of seed bank taxa were absent from aboveground vegetation of PP, MP, and NF stands, respectively. The absence of aboveground species in the seed bank suggested that seeds of most species remained viable on the forest floor for less than one year. Additionally, annual species more frequently reside beneath tree canopies, indicating they must eventually adapt to shaded microenvironments. However, some shade‐intolerant species have lower seed production potential in the understory and most likely contributed to the difference between soil seed bank and understory vegetation. Shade‐intolerant herbaceous species would likely be outcompeted beneath tree canopies before their seedlings could become established.

The seeds of various native tree species were in the seed rain and soil seed bank of the plantations in this study, suggesting that the two seed sources played an important role in native forest regeneration (Douh et al., 2018; Yan, Zhu, Gang, Huang, et al., 2013). Seed rain and seed bank in plantations can assist the efforts of local foresters to promote natural succession and to establish natural plant communities with greater diversity. Accordingly, managers interested in sustainable forestry should consider strategies that take advantage of soil seed banks, such as thinning to reduce litter cover and increase light in the understory. These types of silvicultural practices improve microsite conditions, especially for light‐demanding pioneer species, and should positively influence the regeneration of pure plantations (Yan et al., 2016; Zhu et al., 2010).

Seed dispersal from undisturbed primary forests may be a major seed source for native tree species and play a prominent role in their recolonization (Arrieta & Suarez, 2005; Lee et al., 2005; Sakai et al., 2005; Zamora & Montagnini, 2007). These facts were borne by the finding in the current study indicating that natural forest made a considerable contribution to the native tree species composition in seed rain and soil seed bank of adjacent pure and mixed plantations. In our suited areas, moreover, the abundance of zoochorous and anemochorous seeds found in the seed rain and seed bank serve to emphasize the importance of natural forests as seed source for the colonization of native species in the artificial plantations in southern China. Under the circumstance of habitat fragmentation, seed dispersal by animals is frequently hampered. From this perspective, remnant mature natural forests should be conserved, and plantations should be established nearby to such forests to facilitate natural regeneration.

## 5 CONCLUSIONS

Based on the findings, the following conclusions can be drawn in this study: (1) the number of species in soil seed bank was higher than that in seed rain, (2) the similarity in species composition between seed sources (seed bank and seed rain) and aboveground vegetation was low, (3) both the quantity of seeds in soil seed bank and seed rain was reasonably sufficient, (4) the seed rain and soil seed bank of current plantations play a potentially important role in forest regeneration. We recommend that managers interested in sustainable forestry should take into consideration the presence of existing soil seed banks when developing their management strategies. While these findings are encouraging, we recognize that seedlings can fail to be established because of unfavorable habitat conditions, including a thick litter layer, low light, seed predation, and competition. Accordingly, we recommend that future studies focus on clarifying the environmental factors that limit seedling establishment. Moreover, given the typical protracted time span of natural broadleaf regeneration in plantations, we also recommend accelerating native species establishment by introducing native, late‐successional trees *via* direct seeding or planting of seedlings.

## CONFLICT OF INTEREST

The authors have no conflict of interest.

## AUTHOR CONTRIBUTION

**Bo Liu:** Conceptualization (lead); Data curation (lead); Formal analysis (lead); Funding acquisition (lead); Investigation (equal); Methodology (lead); Project administration (lead); Supervision (lead); Visualization (lead); Writing – original draft (lead); Writing – review & editing (lead). **Qingqing Liu:** Conceptualization (supporting); Data curation (equal); Investigation (equal); Methodology (supporting); Writing – original draft (equal). **Chenxi Zhu:** Conceptualization (supporting); Data curation (equal); Formal analysis (supporting); Investigation (lead); Methodology (equal). **Zhigang Liu:** Investigation (equal). **Zhijun Huang:** Investigation (equal); Methodology (supporting). **Mulualem Tigabu:** Writing – review & editing (equal). **Zongming He:** Investigation (equal). **Yuhui Liu:** Investigation (supporting). **Zhengning Wang:** Conceptualization (equal); Data curation (lead); Formal analysis (lead); Funding acquisition (lead); Investigation (equal); Methodology (lead); Project administration (equal); Supervision (lead); Visualization (equal); Writing – original draft (equal); Writing – review & editing (equal).

## Supporting information

Appendix S1‐S3

## Data Availability

Analyses reported in this article can be reproduced using the data available on Dryad here: https://doi.org/10.5061/dryad.ghx3ffbpx
