# Supplementary material for: Seed rain and soil seed bank in Chinese fir plantations and an adjacent natural forest in southern China: Implications for the regeneration of native species
Source: Ecol Evol. 2022 Jan 26;12(1):e8539. doi: 10.1002/ece3.8539 (PMC8796942; doi:10.1002/ece3.8539)
Supplement: Supplementary file 1 — Appendix S1‐S3 [file ECE3-12-e8539-s001.docx]

**Appendix S1**. Floristic composition and life-forms of soil seed bank and above-ground vegetation of pure Chinese fir plantation (PP).

| Species | Life form | Species performance | | |
| --- | --- | --- | --- | --- |
|  |  | seed bank only | seed bank and vegetation | vegetation only |
| *Helicia cochinchinensis* | Tree | * |  |  |
| *Trema tomentosa* | Shrub | * |  |  |
| *Mallotus apelta* | Shrub | * |  |  |
| *Aristolochia obliqua* | Shrub | * |  |  |
| *Geranium wilfordii* | Herb | * |  |  |
| *Digitaria fibrosa* | Herb | * |  |  |
| *Ageratum conyzoides* | Herb | * |  |  |
| *Conyza japonica* | Herb | * |  |  |
| *Cyperus rotundus* | Herb | * |  |  |
| *Daphniphyllum oldhami* | Tree |  | * |  |
| *Lithocarpus glaber* | Tree |  | * |  |
| *Castanopsis carlesii* | Tree |  | * |  |
| *Schima superba* | Tree |  | * |  |
| *Machilus pauhoi* | Tree |  | * |  |
| *Cunninghamia lanceolata* | Tree |  | * |  |
| *Neolitsea aurata* var*. chekiangensis* | Tree |  | * |  |
| *Toxicodendron succedaneum* | Tree |  | * |  |
| *Sapium discolor* | Tree |  | * |  |
| *Alangium kurzii* | Tree |  | * |  |
| *Diospyros morrisiana* | Tree |  | * |  |
| *Melastoma dodecandrum* | Shrub |  | * |  |
| *Rhododendron henryi* | Shrub |  | * |  |
| *Lygodium japonicum* | Herb |  | * |  |
| *Gahnia tristis* | Herb |  | * |  |
| *Alpinia chinensis* | Herb |  | * |  |
| *Stenoloma chusanum* | Herb |  | * |  |
| *Neolitsea cambodiana var. glabra* | Tree |  |  | * |
| *Castanopsis kawakamii* | Tree |  |  | * |
| *Liquidambar formosana Hance* | Tree |  |  | * |
| *Litsea subcoriacea* | Tree |  |  | * |
| *Machilus thunbergii* | Tree |  |  | * |
| *Phoebe zhennan* | Tree |  |  | * |
| *Elaeocarpus sylvestris* | Tree |  |  | * |
| *Mallotus lianus* | Tree |  |  | * |
| *Symplocos sumuntia* | Tree |  |  | * |
| *Photinia prunifolia* | Tree |  |  | * |

To be continued…

**Appendix S1 Continued**

| Species | Life form | Species performance | | |
| --- | --- | --- | --- | --- |
|  |  | seed bank only | seed bank and vegetation | vegetation only |

| *Clerodendrum canescens* | Shrub |  |  | * |
| --- | --- | --- | --- | --- |

| *Smilax lanceifolia var. opaca* | Shrub |  |  | * |
| --- | --- | --- | --- | --- |
| *Styrax faberi Perk.* | Shrub |  |  | * |
| *Ficus hirta* | Shrub |  |  | * |
| *Maesa japonica* | Shrub |  |  | * |
| *Symplocos lancifolia* | Shrub |  |  | * |
| *Ilex elmerrilliana S. Y. Hu* | Shrub |  |  | * |
| *Ilex dasyphylla* | Shrub |  |  | * |
| *Ilex pernyi Franch.* | Shrub |  |  | * |
| *Pithecellobium lucidum* | Shrub |  |  | * |
| *Ilex pubescens Hook. et Arn.* | Shrub |  |  | * |
| *Callicarpa kochiana* | Shrub |  |  | * |
| *Ilex triflora Bl.* | Shrub |  |  | * |
| *Randia cochinchinensis* (Lour.) *Merr.* | Shrub |  |  | * |
| *Litsea cubeba* | Shrub |  |  | * |
| *Rubus corchorifolius* | Shrub |  |  | * |
| *Ardisia punctata* | Shrub |  |  | * |
| *Ilex formosana* | Shrub |  |  | * |
| *Embelia rudis* | Shrub |  |  | * |
| *Eurya nitida Korthals* | Shrub |  |  | * |
| *Eurya loquiana Dunn* | Shrub |  |  | * |
| *Lindera communis Hemsl.* | Shrub |  |  | * |
| *Rubus reflexus* | Shrub |  |  | * |
| *Mussaenda pubescens* | Shrub |  |  | * |
| *Embelia longifolia* | Shrub |  |  | * |
| *Camellia japonica* | Shrub |  |  | * |
| *Elaeagnus pungens Thunb.* | Shrub |  |  | * |
| *Tarenna mollissima* | Shrub |  |  | * |
| *Clausena excavata Burm.f.* | Shrub |  |  | * |
| *Symplocos lancifolia Sieb. et Zucc.* | Shrub |  |  | * |
| *Millettia dielsiana Harms* | Shrub |  |  | * |
| *Maesa japonica* (Thunb.) *Moritzi* | Shrub |  |  | * |
| *Neolitsea brevipes H. W. Li* | Shrub |  |  | * |
| *Millettia reticulata* | Vine |  |  | * |
| *Ampelopsis grossedentata* | Vine |  |  | * |
| *Millettia dielisana Harms ex Diels* | Vine |  |  | * |
| *Fissistigma oldhamii* | Vine |  |  | * |

To be continued…

**Appendix S1 Continued**

| Species | Life form | Species performance | | |
| --- | --- | --- | --- | --- |
|  |  | seed bank only | seed bank and vegetation | vegetation only |
| *Woodwardia japonica* (L. f.) *Sm.* | Herb |  |  | * |
| *Lophatherum gracile* | Herb |  |  | * |
| *Dryopteris fuscipes* | Herb |  |  | * |
| *Allantodia metteniana* | Herb |  |  | * |
| *Parathelypteris glanduligera* (Kze.) *Ching* | Herb |  |  | * |
| *Calamus thysanolepis* | Herb |  |  | * |
| *Adiantum flabellulatum* | Herb |  |  | * |
| *Selaginella doederleinii* | Herb |  |  | * |
| *Lindsaea orbiculata* (Lamarck) *Mett. ex Kuhn* | Herb |  |  | * |
| *Blechnum orientale* | Herb |  |  | * |

**Appendix S2**. Floristic composition and life-forms of soil seed bank and above-ground vegetation of Mixed Chinese fir–broadleaf plantation (MP).

| Species | Life form | Species performance | | |
| --- | --- | --- | --- | --- |
|  |  | seed bank only | seed bank and vegetation | vegetation only |

| *Helicia cochinchinensis* | Tree | * |  |  |
| --- | --- | --- | --- | --- |
| *Alangium kurzii* | Tree | * |  |  |
| *Trema tomentosa* | Shrub | * |  |  |
| *Diploclisia affinis* | Shrub | * |  |  |
| *Aristolochia obliqua* | Shrub | * |  |  |
| *Mallotus apelta* | Shrub | * |  |  |
| *Ampelopsis grossedentata* | Vine | * |  |  |
| *Geranium wilfordii* | Herb | * |  |  |
| *Oplismenus undulatifolius* | Herb | * |  |  |
| *Echinochloa hispidula* | Herb | * |  |  |
| *Digitaria fibrosa* | Herb | * |  |  |
| *Ageratum conyzoides* | Herb | * |  |  |
| *Conyza japonica* | Herb | * |  |  |
| *Adiantum capillus-veneris* | Herb | * |  |  |
| *Cyperus rotundus* | Herb | * |  |  |
| *Daphniphyllum oldhami* | Tree |  | * |  |
| *Lithocarpus glaber* | Tree |  | * |  |
| *Diospyros morrisiana* | Tree |  | * |  |
| *Castanopsis carlesii* | Tree |  | * |  |
| *Schima superba* | Tree |  | * |  |
| *Sapium discolor* | Tree |  | * |  |
| *Cunninghamia lanceolata* | Tree |  | * |  |
| *Machilus pauhoi* | Tree |  | * |  |
| *Toxicodendron succedaneum* | Tree |  | * |  |
| *Neolitsea aurata var. chekiangensis* | Tree |  | * |  |
| *Melastoma dodecandrum* | Shrub |  | * |  |
| *Litsea cubeba* | Shrub |  | * |  |
| *Rhododendron henryi* | Shrub |  | * |  |
| *Rubus reflexus* | Shrub |  | * |  |
| *Lygodium japonicum* | Herb |  | * |  |
| *Gahnia tristis* | Herb |  | * |  |
| *Alpinia chinensis* | Herb |  | * |  |
| *Stenoloma chusanum* | Herb |  | * |  |
| *Zanthoxylum myriacanthum Wall. ex Hook.f.* | Tree |  |  | * |
| *Castanopsis kawakamii* | Tree |  |  | * |
| *Mallotus lianus* | Tree |  |  | * |

To be continued…

**Appendix S2 Continued**

| Species | Life form | Species performance | | |
| --- | --- | --- | --- | --- |
|  |  | seed bank only | seed bank and vegetation | vegetation only |

| *Symplocos lancifolia* | Tree |  |  | * |
| --- | --- | --- | --- | --- |
| *Litsea subcoriacea* | Tree |  |  | * |
| *Machilus thunbergii* | Tree |  |  | * |
| *Litsea greenmaniana* | Tree |  |  | * |
| *Syzygium buxifolium* | Tree |  |  | * |
| *Symplocos laurina* (Retz.) *Wall.* | Tree |  |  | * |
| *Alangium kurzii* | Tree |  |  | * |
| *Choerospondias axillaris* | Tree |  |  | * |
| *Phoebe zhennan* | Tree |  |  | * |
| *Alniphyllum fortunei* (Hemsl.) *Park.* | Tree |  |  | * |
| *Elaeocarpus sylvestris* | Tree |  |  | * |
| *Symplocos sumuntia* | Tree |  |  | * |
| *Castanopsis fargesii Franch.* | Tree |  |  | * |
| *Clerodendrum canescens* | Shrub |  |  | * |
| *Smilax lanceifolia var. opaca* | Shrub |  |  | * |
| *Tarenna mollissima* | Shrub |  |  | * |
| *Styrax faberi Perk.* | Shrub |  |  | * |
| *Ficus hirta* | Shrub |  |  | * |
| *Maesa japonica* | Shrub |  |  | * |
| *Tricalysia dubia* (Lindl.) *Ohwi* | Shrub |  |  | * |
| *Elaeagnus pungens Thunb.* | Shrub |  |  | * |
| *Ilex dasyphylla* | Shrub |  |  | * |
| *Adinandra millettii* | Shrub |  |  | * |
| *Ilex pernyi Franch.* | Shrub |  |  | * |
| *Ilex pubescens Hook. et Arn.* | Shrub |  |  | * |
| *Callicarpa kochiana* | Shrub |  |  | * |
| *Randia cochinchinensis* | Shrub |  |  | * |
| *Ardisia punctata* | Shrub |  |  | * |
| *Ilex formosana* | Shrub |  |  | * |
| *Embelia rudis* | Shrub |  |  | * |
| *Eurya japonica Thunb* | Shrub |  |  | * |
| *Eurya loquiana Dunn* | Shrub |  |  | * |
| *Lindera communis Hemsl.* | Shrub |  |  | * |
| *Mussaenda pubescens* | Shrub |  |  | * |
| *Embelia longifolia* | Shrub |  |  | * |
| *Millettia reticulata* | Vine |  |  | * |

To be continued…

**Appendix S2 Continued**

| Species | Life form | Species performance | | |
| --- | --- | --- | --- | --- |
|  |  | seed bank only | seed bank and vegetation | vegetation only |

| *Ampelopsis grossedentata* | Vine |  |  | * |
| --- | --- | --- | --- | --- |
| *Millettia dielisana Harms ex Diels* | Vine |  |  | * |
| *Conyza japonica* | Herb |  |  | * |
| *Lophatherum gracile* | Herb |  |  | * |
| *Woodwardia japonica* | Herb |  |  | * |
| *Allantodia metteniana* | Herb |  |  | * |
| *Cibotium barometz* | Herb |  |  | * |
| *Calamus thysanolepis* | Herb |  |  | * |
| *Adiantum flabellulatum* | Herb |  |  | * |
| *Lindsaea orbiculata* | Herb |  |  | * |
| *Blechnum orientale* | Herb |  |  | * |
| *Neolitsea cambodiana var. glabra* | Herb |  |  | * |

**Appendix S3**. Floristic composition and life-forms of soil seed bank and above-ground vegetation of natural broadleaf forest (NF).

| Species | Life form | Species performance | | |
| --- | --- | --- | --- | --- |
|  |  | seed bank only | seed bank and vegetation | vegetation only |

| *Sapium discolor* | Tree | * |  | |  |
| --- | --- | --- | --- | --- | --- |
| *Cunninghamia lanceolata* | Tree | * |  | |  |
| *Mallotus lianus* | Tree | * |  | |  |
| *Mallotus apelta* | Shrub | * |  | |  |
| *Aristolochia obliqua* | Shrub | * |  | |  |
| *Ampelopsis grossedentata* | Vine | * |  | |  |
| *Lygodium japonicum* | Herb | * |  | |  |
| *Ageratum conyzoides* | Herb | * |  | |  |
| *Geranium wilfordii* | Herb | * |  | |  |
| *Blechnum orientale* | Herb | * |  | |  |
| *Cyperus rotundus* | Herb | * |  | |  |
| *Adiantum capillus-veneris* | Herb | * |  | |  |
| *Daphniphyllum oldhami* | Tree |  | | * |  |
| *Castanopsis carlesii* | Tree |  | | * |  |
| *Schima superba* | Tree |  | | * |  |
| *Machilus pauhoi* | Tree |  | | * |  |
| *Toxicodendron succedaneum* | Tree |  | | * |  |
| *Trema tomentosa* | Shrub |  | | * |  |
| *Rhododendron henryi* | Shrub |  | | * |  |
| *Gahnia tristis* | Herb |  | | * |  |
| *Alpinia chinensis* | Herb |  | | * |  |
| *Neolitsea cambodiana var. glabra* | Tree |  | |  | * |
| *Castanopsis kawakamii* | Tree |  | |  | * |
| *Ilex purpurea Hassk.* | Tree |  | |  | * |
| *Garcinia multiflora Champ. ex Benth.* | Tree |  | |  | * |
| *Tsoongiodendron odorum Chun* | Tree |  | |  | * |
| *Symplocos lancifolia* | Tree |  | |  | * |
| *Photinia glabra* (Thunb.) *Maxim.* | Tree |  | |  | * |
| *Litsea subcoriacea Yang et P. H. Huang var. subcori* | Tree |  | |  | * |
| *Machilus thunbergii Sieb.et Zucc.* | Tree |  | |  | * |
| *Cinnamomum austro-sinense* | Tree |  | |  | * |
| *Litsea greenmaniana* | Tree |  | |  | * |
| *Syzygium buxifolium Hook. et Arn* | Tree |  | |  | * |
| *Symplocos laurina* (Retz.) *Wall.* | Tree |  | |  | * |
| *Michelia macclurei Dandy* | Tree |  | |  | * |

To be continued…

**Appendix S3 Continued**

| Species | Life form | Species performance | | |
| --- | --- | --- | --- | --- |
|  |  | seed bank only | seed bank and vegetation | vegetation only |

| *Lithocarpus glaber* | Tree |  |  | * |
| --- | --- | --- | --- | --- |
| *Ormosia xylocarpa* | Tree |  |  | * |
| *Phoebe zhennan* | Tree |  |  | * |
| *Cyclobalanopsis glauca* (Thunb.) *Oerst.* | Tree |  |  | * |
| *Elaeocarpus sylvestris* | Tree |  |  | * |
| *Castanopsis fargesii Franch.* | Tree |  |  | * |
| *Photinia prunifolia* | Tree |  |  | * |
| *Neolitsea cambodiana* | Tree |  |  | * |
| *Neolitsea aurata var. chekiangensis* | Tree |  |  | * |
| *Clerodendrum canescens* | Shrub |  |  | * |
| *Melastoma dodecandrum* | Shrub |  |  | * |
| *Smilax lanceifolia var. opaca* | Shrub |  |  | * |
| *Styrax faberi Perk.* | Shrub |  |  | * |
| *Maesa japonica* | Shrub |  |  | * |
| *Tricalysia dubia* (Lindl.) *Ohwi* | Shrub |  |  | * |
| *Fissistigma oldhamii* | Shrub |  |  | * |
| *Ilex elmerrilliana S. Y. Hu* | Shrub |  |  | * |
| *Ilex dasyphylla* | Shrub |  |  | * |
| *Adinandra millettii* (Hook. et Arn.) *Benth. et Hook. f. ex Hance* | Shrub |  |  | * |
| *Ilex pernyi Franch.* | Shrub |  |  | * |
| *Rhododendron latoucheae Franch.* | Shrub |  |  | * |
| *Ilex pubescens Hook. et Arn.* | Shrub |  |  | * |
| *Ilex triflora Bl.* | Shrub |  |  | * |
| *Randia cochinchinensis* (Lour.) *Merr.* | Shrub |  |  | * |
| *Litsea cubeba* | Shrub |  |  | * |
| *Ardisia punctata* | Shrub |  |  | * |
| *Ilex formosana* | Shrub |  |  | * |
| *Ficus formosana Maxim.* | Shrub |  |  | * |
| *Embelia rudis* | Shrub |  |  | * |
| *Eurya japonica Thunb* | Shrub |  |  | * |
| *Eurya loquiana Dunn* | Shrub |  |  | * |
| *Euonymus euscaphis Hand.Mazz.* | Shrub |  |  | * |
| *Mussaenda pubescens* | Shrub |  |  | * |
| *Embelia longifolia* | Shrub |  |  | * |
| *Tetrastigma hemsleyanum Diels et Gilg* | Vine |  |  | * |
| *Millettia reticulata* | Vine |  |  | * |

To be continued…

**Appendix S3 Continued**

| Species | Life form | Species performance | | |
| --- | --- | --- | --- | --- |
|  |  | seed bank only | seed bank and vegetation | vegetation only |

| *Millettia dielisana Harms ex Diels* | Vine |  |  | * |
| --- | --- | --- | --- | --- |
| *Sarcandra glabra* (Thunb.) *Nakai* | Herb |  |  | * |
| *Tainia dunnii* | Herb |  |  | * |
| *Woodwardia japonica* (L. f.) *Sm.* | Herb |  |  | * |
| *Allantodia metteniana* | Herb |  |  | * |
| *Calamus thysanolepis* | Herb |  |  | * |
| *Lindsaea orbiculata* (Lamarck) *Mett. ex Kuhn* | Herb |  |  | * |
